# Milk Fat Globule Membrane Supplementation in Formula Modulates the Neonatal Gut Microbiome and Normalizes Intestinal Development

**DOI:** 10.1038/srep45274

**Published:** 2017-03-28

**Authors:** Ganive Bhinder, Joannie M. Allaire, Cyrielle Garcia, Jennifer T. Lau, Justin M. Chan, Natasha R. Ryz, Else S. Bosman, Franziska A. Graef, Shauna M. Crowley, Larissa S. Celiberto, Julia C. Berkmann, Roger A. Dyer, Kevan Jacobson, Michael G. Surette, Sheila M. Innis, Bruce A. Vallance

**Affiliations:** 1 Division of Gastroenterology, Department of Pediatrics, BC Children’s Hospital and the University of British Columbia, Vancouver, BC, Canada; 2 Nutrition and Metabolism Research Program, Department of Pediatrics, BC Children’s Hospital and the University of British Columbia, Vancouver, BC, Canada; 3 Department of Biochemistry and Biomedical Sciences, McMaster University, Hamilton, ON, Canada; 4 Department of Medicine, McMaster University, Hamilton, ON, Canada

## Abstract

Breast milk has many beneficial properties and unusual characteristics including a unique fat component, termed milk fat globule membrane (MFGM). While breast milk yields important developmental benefits, there are situations where it is unavailable resulting in a need for formula feeding. Most formulas do not contain MFGM, but derive their lipids from vegetable sources, which differ greatly in size and composition. Here we tested the effects of MFGM supplementation on intestinal development and the microbiome as well as its potential to protect against *Clostridium difficile* induced colitis. The pup-in-a-cup model was used to deliver either control or MFGM supplemented formula to rats from 5 to 15 days of age; with mother’s milk (MM) reared animals used as controls. While CTL formula yielded significant deficits in intestinal development as compared to MM littermates, addition of MFGM to formula restored intestinal growth, Paneth and goblet cell numbers, and tight junction protein patterns to that of MM pups. Moreover, the gut microbiota of MFGM and MM pups displayed greater similarities than CTL, and proved protective against *C. difficile* toxin induced inflammation. Our study thus demonstrates that addition of MFGM to formula promotes development of the intestinal epithelium and microbiome and protects against inflammation.

During gestation, the gastrointestinal (GI) tract is immature, and possesses limited functions as most nutrients are obtained via placental transfer. Following birth, there is a switch to nutrient acquisition from ingested food, and a corresponding maturation of the intestine. The source, makeup as well as the quantity of these nutrients are important in overall development of the infant, and can act locally in regulating the maturation of the intestine and the makeup of the gut microbes that colonize the neonate’s GI tract^1^,^2^,^3^.

Using rodent and avian models, several groups have explored postnatal intestinal development from birth to one year of age, revealing major changes in epithelial architecture along the GI tract^4^,^5^,^6^,^7^,^8^. Within the small intestine, crypt depths and villus lengths significantly increase during the first month, and the number of crypts within the large intestine doubles^4^. Others have reported increases in innervation of submucosal ganglia^5^, changes in localization of epithelial tight junction (TJ) proteins^6^, and significant increases in goblet cell (GC) numbers and mucins^7^,^8^ during the neonatal period. Proper intestinal development facilitates the overall development of the neonate, but is also important in providing appropriate defense against noxious stimuli. This is particularly important for premature babies who are born with an immature GI tract, leaving them highly susceptible to infections and Necrotizing Enterocolitis (NEC), a leading cause of GI morbidity and mortality in premature infants^9^.

Neonate development is fueled by breast milk, the ideal nutrient source during this stage of life. Further, breastfeeding has been reported to lower risk of infection and diarrhea^10^,^11^,^12^,^13^, and to protect against development of asthma, allergies and immune mediated diseases^3^,^11^,^14^,^15^. Its protective functions have been attributed to antibodies, enzymes (e.g. lysozyme, alkaline phosphatase) and growth factors (e.g. transforming growth factor-β and insulin like growth factor)^16^,^17^. Unfortunately, breast milk is often unavailable in sufficient quantities, if at all, to satisfy the nutrient requirements of newborns, particularly with premature births. As a result, formula feeding has taken on a significant role in neonatal development and health care^18^. Therefore, we and others have proposed that optimal formula composition should match that of breast milk as closely as possible^1^,^19^.

The lipid fraction of breast milk, representing a major energy source for the newborn, is composed of a triacylglycerol (TAG) core surrounded by a unique triple membrane structure: the milk fat globule membrane (MFGM)^19^,^20^. MFGM, derived from the mammary gland epithelium, is composed primarily of polar lipids with interspersed membrane-bound proteins, glycoproteins, enzymes and cholesterol resulting in a bioactive molecule that likely confers some of the protective effects of breast milk^1^,^2^. Most available infant formulas do not contain MFGM, but rather derive their lipids from vegetable sources, which differ greatly in size (1/10^th^ the diameter) and composition^19^. Specifically, MFGM and vegetable derived lipids differ in TAG composition and internal structure, while the bioactive molecules present in MFGM are largely absent from formula lipids^19^. Recent breakthroughs in manufacturing technologies permit the concentration of bovine MFGM, making it feasible to add into infant formula.

Previous studies examining MFGM supplementation to piglets and human infants have predominantly focused on neurodevelopment, with its addition increasing cognitive scores compared to control formula, and similar to those of breastfed infants^21^,^22^,^23^. Interestingly, a study examining the incidence of acute otitis media (AOM) and antipyretic use in human infants found that MFGM supplementation in formula resulted in decreased AOM and fewer days with fever compared to infants consuming control formula^24^. Additional studies using rodent models (>6 weeks old) have examined the effects of MFGM supplementation on infection and inflammation *in vivo* and *in vitro* 25,26,27. Components of MFGM display *in vitro* bactericidal activity against several foodborne pathogens, including *Campylobacter jejuni, Salmonella enteriditis*, and *Listeria monocytogenes*^25^. *In vivo*, rats supplemented with MFGM and then infected with *L. monocytogenes* were protected against pathogen colonization and translocation^25^. During lipopolysaccharide-induced systemic inflammation in mice, MFGM supplementation significantly reduced gut barrier disruption and inflammatory cytokines^27^. Finally, in a rat model of dimethylhydrazine induced colon cancer, MFGM offered protection from aberrant crypt foci development, as compared to diets containing corn oil as their fat source^26^.

Given the aforementioned protective effects of MFGM, we hypothesized that its supplementation in formula might prove beneficial within the developing intestine. To test this, we utilized the unique pup-in-a-cup model of artificial rearing, allowing exclusive formula feeding of rat pups starting at postnatal (pn) day 5. Pups were provided either control (CTL) formula, with fat derived exclusively from vegetable sources, or with an identical formula with MFGM comprising part of the fat component. Rat pups left with mothers, and fed mother’s milk (MM), served as positive controls. Interestingly, rats fed CTL formula showed delayed intestinal growth as compared to MM fed pups at pn day 15. Notably, the addition of MFGM normalized most readouts to the levels seen in MM littermates, including intestinal crypt depths, epithelial cell proliferation, makeup of intestinal epithelial cell (IEC) subsets, and similar make-up of intestinal microbes at the phylum level. Lastly, upon challenge with *Clostridium difficile* toxins, MFGM supplementation afforded significant protection from mucosal damage as compared to CTL rat pups. Our study thus demonstrates that MFGM supplementation promotes intestinal epithelial and microbiome development and confers significant protection against noxious inflammatory stimuli.

## Results

### Rat Pup Growth and Gross Intestinal Characteristics

**Figure 1:**
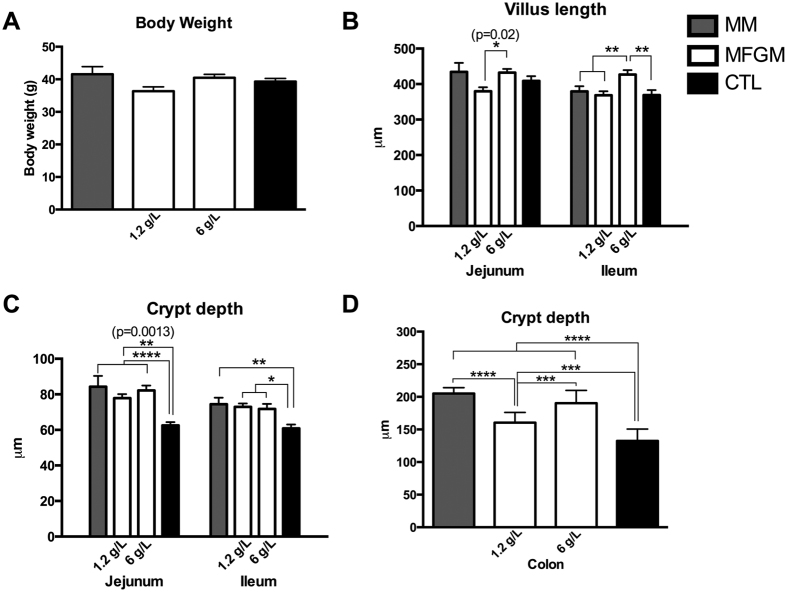
MFGM supplementation in formula normalizes intestinal architecture in a dose dependent manner without affecting body weight at pn day 15. (**A**) Body weights (g) of rat pups in the 4 different diet groups were similar after ten days of supplementation. n ≥ 10 (**B**) Villus lengths, measured in μm, in the jejunum (left) and ileum (right). Dose dependent increases in length in the MFGM supplemented group were observed. n = 8–10. Crypt depth in the jejunum (left) and ileum (right) (**C**) and colon (**D**) with CTL formula fed pups showing significantly decreased depths compared to all other groups. n = 8–10. The graphed data presented are the mean ± SEM, analyzed by One-way ANOVA followed by Tukey’s multiple comparisons test. *p < 0.05, **p < 0.005, ***p < 0.0005, ****p < 0.0001.

To determine if MFGM supplementation altered overall growth of the animals, pups were weighed daily beginning at pn day 5 until day 15. All groups displayed similar body weight after 10 days of supplementation (Fig. 1A). In addition, average daily weight gain was similar between the three formula groups (Supplementary Fig. 1). Upon euthanization at pn day 15, the overall length of the small intestine and liver weights were similar between all groups (Supplementary Fig. 1).

### Impact of MFGM on Intestinal Epithelial Architecture & Barrier

As villus lengths and crypt depths are overt markers of intestinal health, they were next assessed to determine if intestinal architecture was impacted by MFGM supplementation. In the jejunum, CTL formula, MM and 6 g/L MFGM pups displayed similar villus lengths, with MFGM fed pups displaying a dose dependent increase in villus lengths in both the jejunum and the ileum (Fig. 1B). In the ileum, CTL formula, MM and 1.2 g/L MFGM pups displayed similar villus lengths, while 6 g/L MFGM supplementation resulted in significantly longer villi at this site (Fig. 1B).

Although the CTL formula group showed normal villus lengths, they displayed significantly shorter crypt depths as compared to MM and both MFGM groups in the jejunum and ileum (Fig. 1C). In the distal colon, CTL formula pups again displayed significantly shorter crypts than MM and both MFGM groups (Fig. 1D). A dose dependent increase in distal colonic crypt depths was also recorded in the MFGM fed pups, where 6 g/L supplementation restored crypt depths to levels similar to MM pups. As these assessments noted greater effects on intestinal villus and crypt architecture following 6 g/L MFGM supplementation, subsequent analysis focused on this dose.

**Figure 2:**
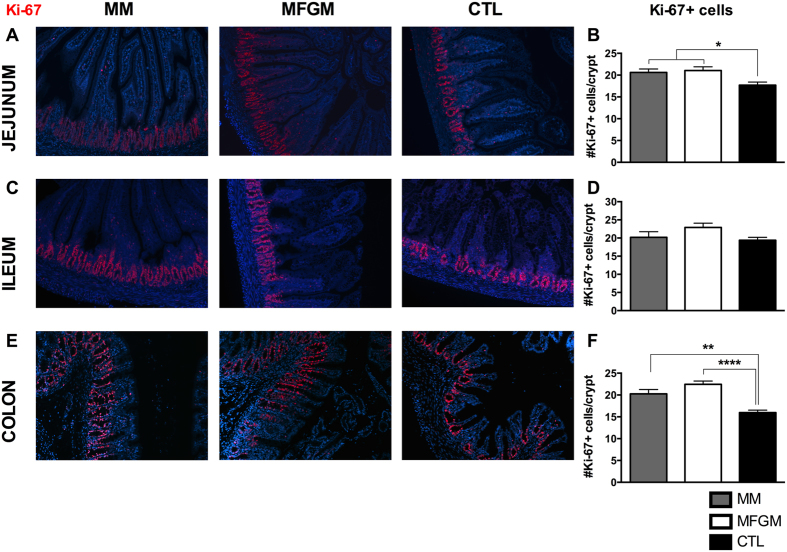
Intestinal proliferation at pn day 15 is increased in formula fed pups with 6 g/L MFGM supplementation, similar to levels in MM fed pups. Representative images of immunostaining for the proliferation marker Ki-67 (red) and DNA (blue) in the jejunum (**A**), ileum (**C**), and colon (**E**) of MM, 6 g/L MFGM and CTL formula fed pups, with corresponding quantification of number of Ki-67 positive cells per crypt in (**B**,**D** and **F**). n > 6. The graphed data presented are the mean ± SEM, analyzed by one-way ANOVA followed by Tukey’s multiple comparisons test. *p < 0.05, **p < 0.005, ****p < 0.0001. Original Magnification: 200X.

Next, we examined the contribution of IEC proliferation to the changes observed in intestinal architecture by immunostaining tissues collected at pn day 15 for the nuclear proliferation marker Ki-67. In both the jejunum and colon, MFGM and MM fed pups displayed similar Ki-67 positive staining, whereas the CTL pups had significantly fewer positive cells/crypt (Fig. 2A–F). In the ileum, similar numbers of Ki-67 positive cells/crypt were recorded in all three groups (Fig. 2C,D).

**Figure 3:**
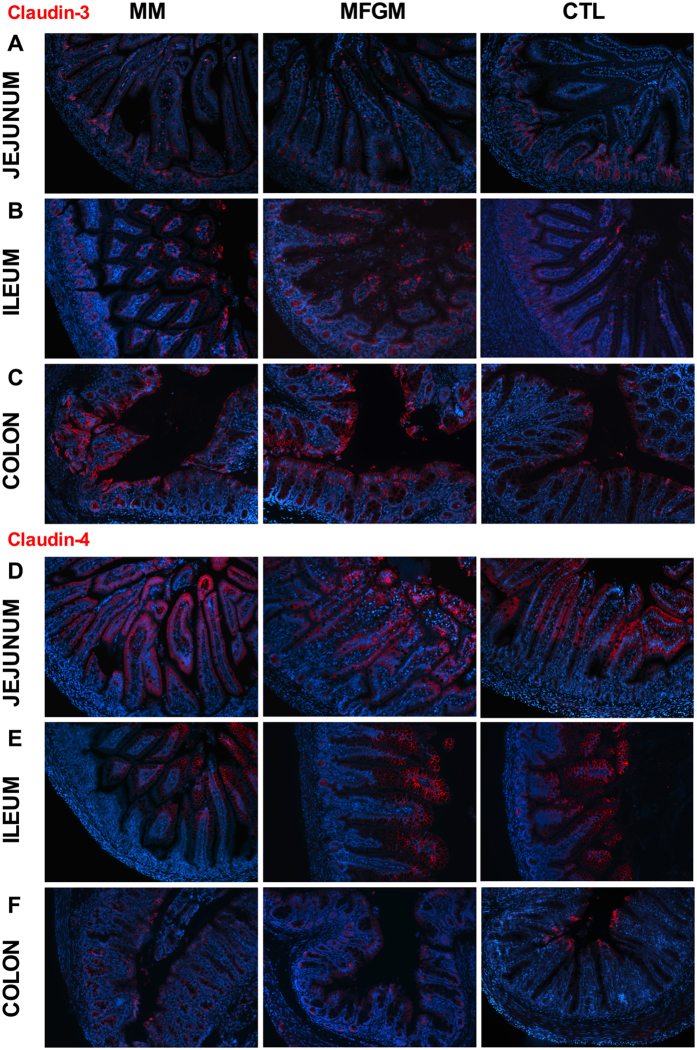
Localization of barrier proteins, Claudin-3 and Claudin-4, are similar in MM and 6 g/L MFGM pups compared to CTL formula fed littermates. Representative images from jejunal (**A**,**D**), ileal (**B**,**E**) and colonic (**C**,**F**) tissues of pn day 15 MM, 6 g/L MFGM and CTL formula fed pups immunostained for the TJ proteins (red) Claudin-3 (**A**–**C**), Claudin-4 (**D**–**F**) and DNA (blue). n = 5–8. Original Magnification: 200X.

To clarify whether formula feeding altered the intestinal epithelial barrier, we next immunostained for the TJ proteins Claudin-3 and Claudin-4. At all three sites (jejunum, ileum and colon), MM and MFGM fed pups displayed similar positive staining for Claudin-3, as compared to its limited staining in CTL formula littermates (Fig. 3A–C). At the small intestinal sites, Claudin-3 was broadly expressed along the entire length of the villi in MM and MFGM pups, whereas its staining was sporadic and limited to the crypts in CTL fed pups (Fig. 3A,B). In the colon, MM and MFGM pups showed greater positive staining along the tops of the crypts compared to CTL fed pups (Fig. 3C). In contrast, Claudin-4 displayed similar positive staining in all three groups at both small intestinal sites (Fig. 3D,E), however within the colon CTL fed pups displayed greater positive staining at the tips of crypts as compared to MM and MFGM groups (Fig. 3F). Claudin-3 and 4 staining was semi-quantified by examining their fluorescence intensity in villi relative to crypts at small intestinal sites and at the top of crypts relative to the rest of the crypts in colonic tissues, confirming the results observed in Fig. 3 (Claudin 3: jejunum: MM = 5.5 ± 1.2 vs. CTL = 0.4 ± 0.2 (p = 0.025), colon: MM = 1.0 ± 0.2 vs. CTL = 0.4 ± 0.1 (p = 0.01), MFGM = 0.9 ± 0.06 vs. CTL (p = 0.02); Claudin-4: colon: MM = 0.2 ± 0.02 vs. CTL = 0.5 ± 0.05 (p < 0.0001), MFGM = 0.3 ± 0.03 vs. CTL (p < 0.0005)) (Supplementary Fig. 1). Finally, to determine whether these differences in expression of TJ proteins had any functional impact, we assessed barrier permeability using the FITC-dextran assay. Interestingly, no significant differences in barrier permeability were observed between the three groups at pn day15 (Supplementary Fig. 1).

### Changes in Intestinal Epithelial Cell Subtypes

Rapid changes in the development of the various IEC subtypes have been documented during early intestinal development in several animal models^7^,^8^,^28^,^29^,^30^. Therefore, we next examined the impact of MFGM supplemented formula on the development of IECs.

#### Paneth Cells

**Figure 4:**
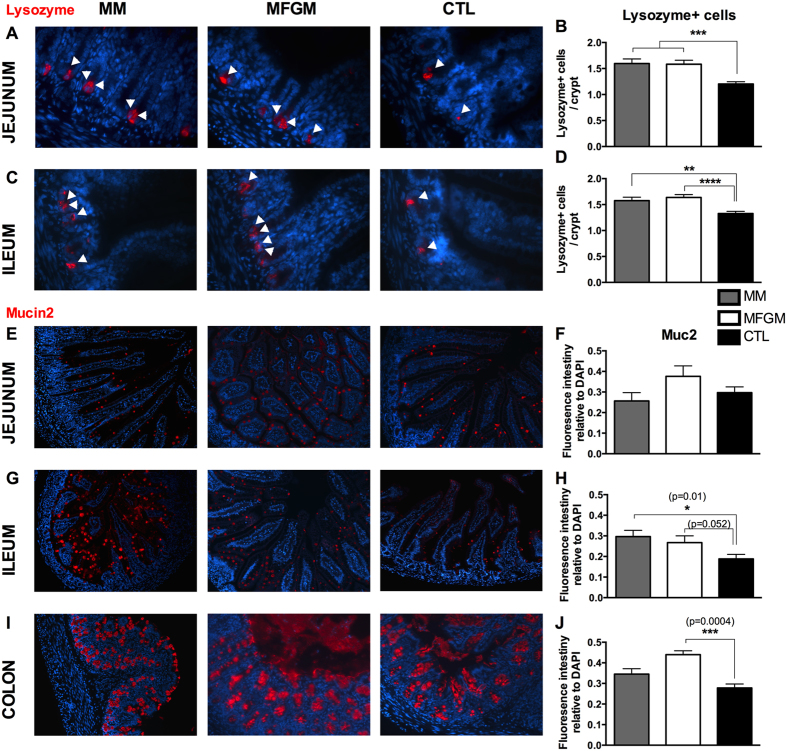
Addition of 6 g/L MFGM to formula normalizes Paneth cell numbers and increases Muc2 positive staining at pn day 15. Representative images of immunostaining for the Paneth cell antimicrobial protein Lysozyme (red) and DNA (blue) in the jejunum (**A**) and ileum (**C**), quantified as number of lysozyme positive cells per crypt in (**B**) and (**D**). White arrowheads indicate lysozyme positive Paneth cells. Representative immunostaining of Muc2 (red), with DNA in blue, in jejunal (**E**), ileal (**G**) and colonic (**I**) tissues of MM, 6 g/L MFGM and CTL formula fed rat pups. Fluorescence intensity measurements for Muc2 relative to total DNA staining in sections in (**F**,**H** and **J**). n > 6. The graphed data presented are the mean ± SEM, analyzed by Kruskal-Wallis test (**B**,**D**,**H**,**J**) or One-way ANOVA (**F**) followed by multiple comparisons tests. *p = 0.01, **p < 0.005, ***p < 0.0005, ****p < 0.0001. Original Magnification: 630X (**A**,**C**), 200X (**E**,**G**,**I**).

PCs are a secretory cell type found at the base of crypts in the small intestine and a major source of antimicrobial peptides, such as defensins, cathelicidins and lysozyme. During murine intestinal development, PCs have been found in small intestinal tissues by pn day 7, with rapid increases observed in following weeks^28^. We examined PC numbers in the jejunum and ileum of our pn day 15 rat pups by immunostaining for lysozyme. At both small intestinal sites, MM and MFGM supplemented pups displayed similar numbers of lysozyme-positive cells/crypt, whereas significantly fewer lysozyme-positive cells/crypt were identified in CTL formula tissues (Fig. 4A–D).

#### Goblet Cells

GCs are the most numerous secretory cell type found within the intestinal epithelium, where they synthesize and secrete mucins, such as Muc2, and other protective bioactive molecules^31^,^32^,^33^,^34^. GC numbers have also been found to significantly increase within the intestinal epithelium during postnatal development^7^,^8^. We assessed GCs by immunostaining for Muc2, and measuring Muc2 fluorescence intensity relative to DAPI (nuclear stain). Staining in the small intestine showed individual Muc2 positive goblet cells scattered along the villus lengths and within crypts (Fig. 4E and G). Although similar levels of Muc2 positive staining were observed between the three groups in the jejunum, in the ileum, MM tissues displayed significantly more Muc2 positive staining than CTL formula tissues. Interestingly MFGM tissues showed Muc2 levels similar to that seen in MM tissues (Fig. 4E–H). In the colon, Muc2 staining was much greater than in the small intestine with a mean ratio of Muc2/DAPI positive staining for all three groups of 0.16 ± 0.01 in the ileum and 0.30 ± 0.025 in the colon (p < 0.0001, Mann-Whitney test), reflecting the presence of numerous goblet cells. Interestingly, while Muc2 staining was modestly, but not significantly greater in the MM group, as compared to CTL tissues, MFGM tissues displayed significantly increased Muc2 positive staining as compared to both MM and CTL formula groups (Fig. 4I,J).

#### Enteroendocrine Cells

**Figure 5:**
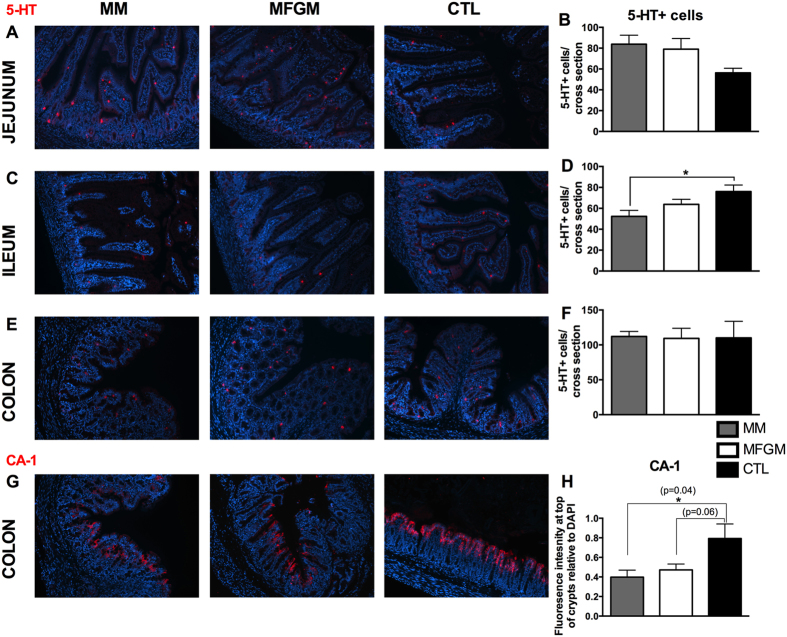
Enteroendocrine numbers and Enterocyte staining are similar in 6 g/L MFGM and MM pups, compared to CTL formula fed littermates. Representative immunostained images for 5-HT (red) in the jejunum (**A**), ileum (**C**) and colon (**E**) to assess enteroendocrine cells with DNA in blue. Similar numbers of enteroendocrine cells per tissue section were counted in the jejunum (**B**) and colon (**F**) of all three groups, with CTL formula fed ileal sections displaying increased 5-HT positive cells (**D**). Representative images of CA-1 (red) and DNA (blue) immunostaining to assess enterocytes in the colon (**G**), with MM and 6 g/L MFGM pups displaying similar staining patterns compared to CTL formula fed pups. (**H**) Fluorescence intensity measurements for CA-1 positive staining relative to DAPI at the surface of colonic crypts. n > 6. The graphed data presented are the mean ± SEM, analyzed by One-way ANOVA followed by Tukey’s multiple comparisons test. *p < 0.05. Original Magnification: 200X.

Enteroendocrine cells in small intestinal and colonic tissues were examined by immunostaining for 5-Hydroxytryptophan (5-HT). In the jejunum, similar amounts of 5-HT-positive cells/tissue cross section were observed in MM and MFGM pups, which was increased, though not significantly, compared to CTL formula pups (Fig. 5A,B). In the ileum this was reversed as the CTL formula fed group carried significantly more 5-HT-positive cells/tissue cross section compared to MM (Fig. 5C,D). In both tissues, the addition of MFGM brought the numbers of 5-HT-positive cells/tissue cross section closer to that seen in the MM pups, although the numbers did not reach significance. Interestingly all three groups displayed similar numbers of 5-HT-positive cells/tissue cross section in the colon (Fig. 5E,F).

#### Enterocytes

Enterocytes were examined at the three intestinal sites by immunostaining for carbonic anhydrase-1 (CA-1), an electrolyte transporter^29^,^30^. At both small intestinal sites, CA-1 staining patterns were quite similar between the three groups (Supplementary Fig. 2). However, in the colon, the CTL formula fed pups displayed a very distinct and thick layer of CA-1 positive staining along the surface of the crypts, whereas MFGM and MM pups displayed patchier positive staining (Fig. 5G and H), potentially reflecting the presence of numerous non-enterocytes (i.e. GCs) in these pups.

### Contribution of Microbiota to Normalization of Intestinal Development

As many studies and reviews have highlighted the importance of intestinal microbes in postnatal intestinal development^1^,^35^,^36^, we next assessed total microbe numbers in the feces of the three groups (Supplementary Fig. 3). Interestingly, MM pups harbored the greatest number of microbes (5.5 × 10^10^ CFU/g feces), at least 10-fold greater than MFGM and CTL pups (p < 0.0001; Supplementary Fig. 3), likely reflecting their continued contact with their mother and her microbiome. CTL pups carried the lowest number of microbes (6.2 × 10^9^ CFU/g feces), with MFGM pups carrying slightly more (9.7 × 10^9^ CFU/g feces) at pn day 15 (Supplementary Fig. 3).

**Figure 6:**
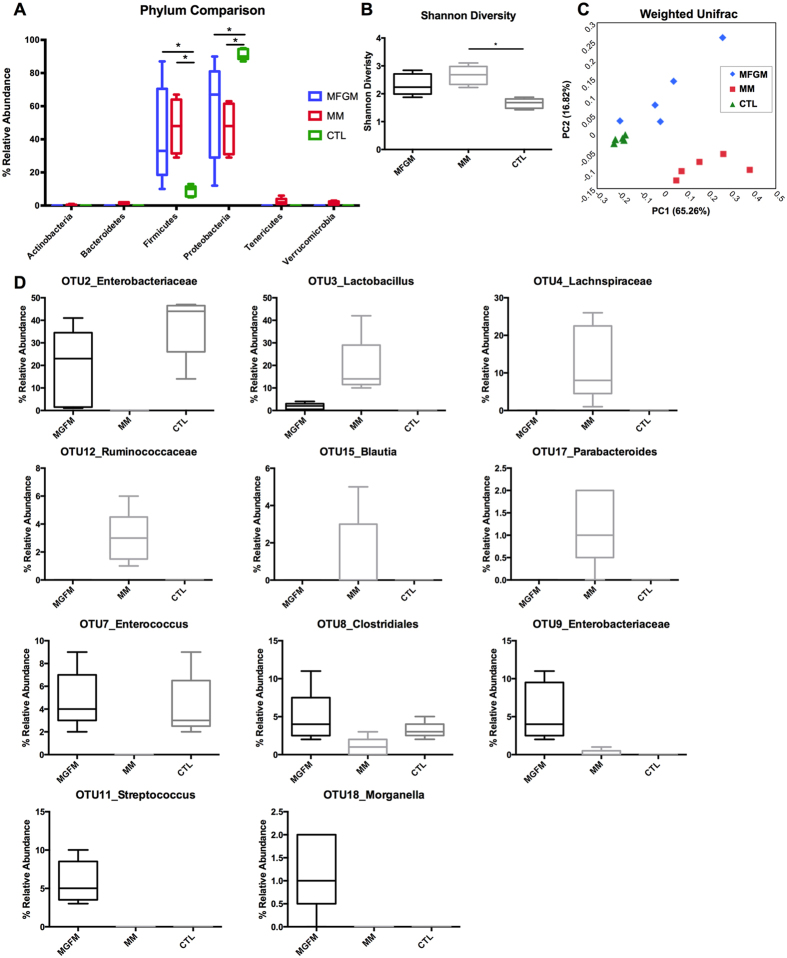
Food source impacts the composition of the intestinal microbiome. Microbial communities in fecal pellets at pn day 15 were assessed by 16S rRNA gene sequencing. (**A**) Phylum level comparison of relative abundance of bacterial taxa revealed similar abundance of Firmicutes and Proteobacteria in MM (blue) and 6 g/L MFGM (red) pups as compared to CTL (green) formula fed pups. Two-way ANOVA followed by Tukey’s multiple comparisons test. (**B**) MM pups harbored microbiota with significantly higher richness and evenness than CTL formula pups (Shannon Diversity index, Kruskal-Wallis with Dunn’s Multiple Comparison) and clustering of MM, 6 g/L MFGM and CTL formula fed pups was significant (**C**) Weighted UniFrac adonis R^2^ = 0.6036, *p* = 0.001. (**D**) LEfSs analysis to determine differences in OTUs (greater than 1% relative abundance) between the three groups, significantly different OTUs are shown. n = 5. *p < 0.05.

To further assess differences in the microbiome we next analyzed their makeup in the three groups using 16S rRNA gene sequencing, finding distinct differences between the three groups. At the phylum level, the MM and MFGM supplemented pups displayed similar abundances of Firmicutes and Proteobacteria, as compared to CTL formula fed littermates who carried significantly lower levels of Firmicutes and higher levels of Proteobacteria (Fig. 6A). However, at the genus level, major differences in taxa between all three groups were detected (Supplementary Fig. 3). Upon examining the diversity within the samples, MFGM and MM pups displayed greater species richness and evenness than the CTL formula group; with CTL formula leading to significantly decreased alpha-diversity compared to MM pups (Fig. 6B). Upon examining beta-diversity between the three groups using Weighted Unifrac analysis of all OTUs, clustering of MM, MFGM and CTL formula pups was found to be significant suggesting the three groups harbored a distinct pattern of taxa from one another (Fig. 6C). Finally, we performed LEfSe analysis to determine which OTUs were significantly different between the three groups (Fig. 6D). Taking into account only the most abundant OTUs (greater than 1% relative abundance), we observed that Enterobacteriaceae were significantly higher in the CTL group, decreased in the MFGM and absent in the MM group. Lactobacilli were significantly greater in the MM group, decreased in the MFGM and absent in the CTL group. Additional OTUs that were significantly higher in the MM group included Lachnospiraceae, Ruminococcaceae, Blautia and Parabacteroides. OTUs that were significantly increased in the MFGM group were Enterococcus, Clostridiales, Streptococcus and Morganella.

We next tested the impact of microbes on intestinal development, using an antibiotic cocktail to deplete the microbes in the MM group, which displayed the greatest abundance and diversity of microbes, as well as in the MFGM formula group. Enumeration of fecal microbe numbers revealed a 10-fold depletion of microbes at pn day 15 in MM pups (4.4 × 10^9^ CFU/g feces) resulting in microbial numbers roughly similar to those observed in untreated CTL and MFGM formula fed pups (p < 0.0005; Supplementary Fig. 3). In contrast, antibiotic treatment of MFGM formula fed pups only depleted intestinal microbes by 45% (from 9.7 × 10^9^ to 5.1 × 10^9^ CFU/g feces) (p < 0.05; Supplementary Fig. 3). Based on the limited depletion seen in the formula fed pups, further analysis regarding the impact of microbiome depletion on intestinal development was focused on the MM antibiotic treated pups.

**Figure 7:**
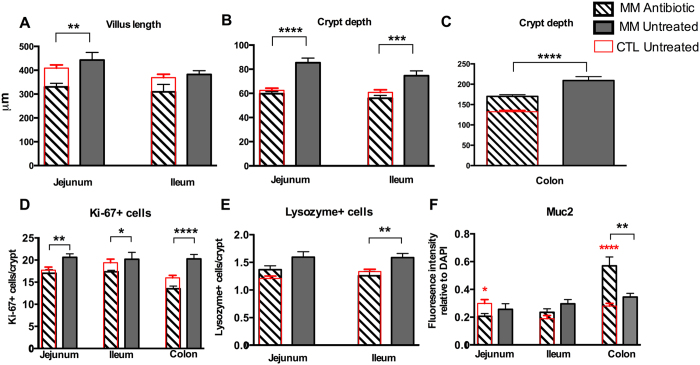
Intestinal development is partially dependent on the presence of intestinal microbes in MM raised pups. (**A**) Villus lengths and (**B**) Crypt depths in the jejunum (left), ileum (right) and colon (**C**) of MM pups treated with an Antibiotic cocktail for 7 days and untreated MM fed pups at pn day 15. Antibiotic treatment led to decreases in villus lengths, and crypt depths in MM fed pups. Quantification of cell proliferation (**D**) as number of Ki-67 positive cells per crypt, Paneth cells (**E**) as Lysozyme positive cells per crypt and (**F**) Muc2 fluorescence intensity relative to DNA in the jejunum, ileum and colon of Antibiotic cocktail treated and untreated MM pups. Antibiotic treatment led to a decrease in cell proliferation at all sites, decreased Paneth cells in the ileum, and an increase in Muc2 intensity in the colon. Measurements of untreated CTL formula fed pups are presented as a reference (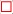). n = 5. The graphed data presented are the mean ± SEM, analyzed by unpaired Student’s t-test. *p < 0.05, **p < 0.005, ***p < 0.0005, ****p < 0.0001.

Notably, antibiotic treatment of MM pups did not cause any significant changes in body weight, or small intestinal/colonic length as compared to untreated MM littermates (Supplementary Fig. 4). To test whether the loss of microbes had any impact on intestinal development, villus lengths in both the jejunum and ileum were measured. They showed a significant decrease in MM pups post-antibiotic treatment as compared to untreated MM littermates (Fig. 7A). Interestingly, although crypt depths were significantly decreased at all three intestinal sites in antibiotic treated MM pups as compared to untreated MM littermates, the depths were similar to, if not higher than, those recorded in untreated CTL formula fed pups (Fig. 7B,C). Similar to untreated MM littermates, untreated MFGM crypt depths were significantly greater than antibiotic treated pups (jejunum: MM antibiotic: 59.7 μm ± 1.7, MFGM: 82.2 μm ± 2.8 (p < 0.0001), ileum: MM antibiotic: 55.9 μm ± 2.4, MFGM: 71.8 μm ± 2.8 (p = 0.0001), colon: MM antibiotic: 170.2 μm ± 4.1, MFGM: 190.2 μm ± 4.6 (p = 0.003), n = 5, Student’s t-test).

We next examined epithelial cell proliferation by again immunostaining intestinal tissues for Ki-67. Antibiotic treatment resulted in significantly fewer Ki-67-positive cells/crypt in MM pups at all three sites when compared to untreated littermates (Fig. 7D). When compared to CTL formula supplemented pups, antibiotic treatment of MM pups resulted in a similar number of Ki-67 positive cells at all three sites (Fig. 7D).

IEC subtypes were again quantified by immunostaining tissue sections, to assess the effects of antibiotic treatment. No differences in PC numbers with antibiotic treatment were noted in the jejunum (Fig. 7E), however, in ileal tissues treated MM pups showed a significant reduction in PCs compared to untreated pups, similar to numbers observed in CTL formula fed pups (Fig. 7E). Assessment of GCs in the jejunum and ileum revealed MM antibiotic treated pups had similar levels of Muc2 fluorescence intensity when compared to untreated MM pups, whereas in the colon MM pups surprisingly displayed an increase in Muc2 intensity after exposure (Fig. 7F).

### MFGM supplementation protects against *Clostridium difficile* toxin induced colitis

**Figure 8:**
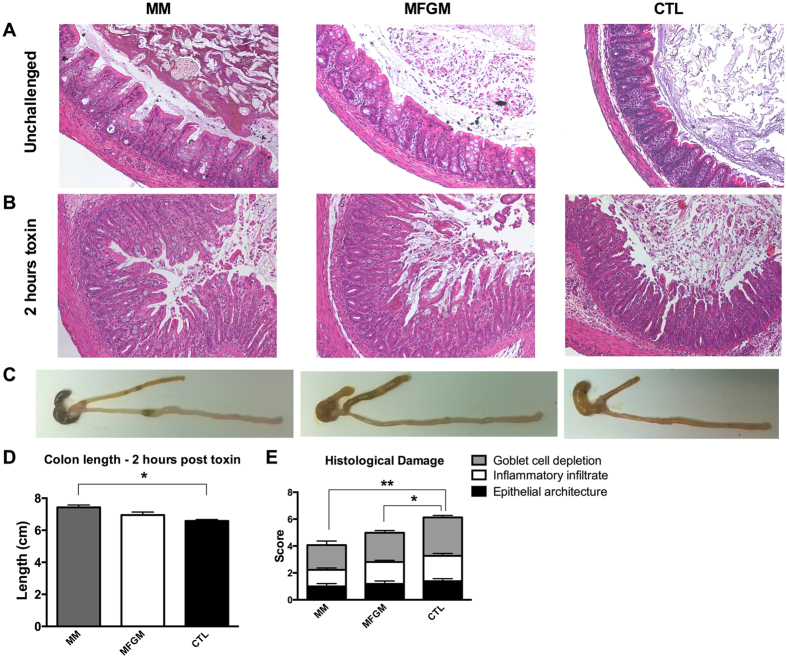
MFGM supplementation protects the formula fed neonate intestine from *C. difficile* toxin induced damage. Representative Hematoxylin & Eosin stained distal colonic sections from MM, 6 g/L MFGM and CTL formula fed pups under (**A**) unchallenged conditions, and (B) after intra-rectal exposure to *C. difficile* toxins TcdA and TcdB for 2 hours at pn day 15. (**C**) Representative macroscopic images of toxin induced colonic shortening and inflammation, quantified in (**D**). (**E**) Comparative histological damage scores after 2 hours toxin exposure in the three groups. n = 6–7. The graphed data presented are the mean ± SEM, analyzed by One-way ANOVA followed Tukey’s multiple comparisons test. *p < 0.05, **p < 0.005. Original Magnification: 200X.

While MFGM supplementation positively impacts the structure and makeup of the neonatal intestinal epithelium, the effect of these changes on overall gut protection was unclear. Neonates are vulnerable to a variety of enteric infections, as well as idiopathic causes of intestinal inflammation such as NEC. Unfortunately there are few models for these conditions that have been established in rat pups. To overcome this, we tested the susceptibility of pups to *C. difficile* toxin induced colitis through intra-rectal challenge, as previously described in adult mouse models^37^,^38^. As in the mouse model of toxin induced colitis, 2 hours of exposure in pn day 15 rat pups resulted in mucosal inflammation as evidenced by inflammatory cell infiltration, submucosal edema, GC depletion and breakdown of epithelial architecture compared to unchallenged littermates (Fig. 8A,B). Furthermore, *C. difficile* toxin exposure led to significant colonic shortening in the formula fed groups as compared to unchallenged groups (Fig. 8C,D), with more pronounced shortening in the toxin treated CTL formula group, significantly more than that in toxin challenged MM pups. Assessment of histological damage by blinded observers revealed significantly greater overall damage scores in the CTL formula group after toxin challenge when compared to both the MFGM and MM pups (Fig. 8E). Thus, supplementation of formula with MFGM yields a state of protection against the colitis caused by *C difficile* toxins.

## Discussion

Breast milk is considered the optimal food source for neonates, however it is not always available to developing infants. Manufacturing formulas that more closely reflect the composition and function of breast milk is therefore of great importance to optimize outcomes among formula fed infants. In this study we tested the effect of formula supplemented with bovine MFGM, which contains similar components to human MFGM, on intestinal development. By utilizing the pup-in-a-cup model, we showed that although CTL formula led to impaired intestinal development as compared to MM fed pups, the addition of MFGM to formula beneficially aligned intestinal readouts to levels similar to those observed with MM. Tissues were examined at pn day 15, as previous studies have reported significant increases in villus lengths and crypt depths by this time point during development^39^,^40^. In addition, due to the size restriction of rat pups in cups, studies utilizing this model have generally terminated on pn days 12–18^39^,^40^,^41^,^42^. As no differences were seen in weight gain between the three diet groups, and the CTL and MFGM formula fed groups were administered identical volumes of formula daily, the differences noted in intestinal mucosal development are not due to differences in the volume of food consumed or to differences in weight gain.

Through histological and immunofluorescence analysis, we found that MFGM supplementation led to similar intestinal mucosal architecture at small intestinal and colonic sites to that seen in MM pups. In addition, MFGM supplementation led to similar numbers of proliferating cells, PCs, and GCs as those observed in MM raised pups. The increase in secretory cells (PCs, GCs) with MFGM supplemented formula compared to CTL is noteworthy as previous studies have reported significantly decreased numbers of these cell types in the resected tissues of children with NEC, as compared to children with other non-inflammatory colonic diseases^43^. In addition, MFGM and MM pups displayed similar TJ protein staining at all intestinal sites, whereas CTL formula fed pups showed altered staining patterns for Claudin-3 at all three sites, and Claudin-4 in the colon compared to the other two groups. Upon assessing the effect of these alterations on barrier function, using the FITC-dextran assay, no differences in permeability were observed; suggesting the differences in TJ protein staining in the CTL formula group did not result in overt barrier disruption under homeostatic conditions.

In addition to serving as an important nutrient source, the composition of breast milk and/or formula can also impact the makeup of the gut microbes that colonize the neonate GI tract^1^,^2^,^3^. As the colon harbors the highest density of intestinal microbes, this may help explain why the effects of MFGM supplementation compared to CTL formula appeared greatest in the colon. In this study, we found that MFGM supplementation of formula altered the makeup of the gut microbiota, resulting in a microbiome that at the phylum level resembled that found in MM pups. In contrast, CTL formula pups harbored a significantly higher percentage of Proteobacteria and a lower percentage of Firmicutes as compared to both MM and MFGM fed pups. These results are similar to what some studies have found for phylum level differences in the microbiota of formula fed versus breastfed infants, with formula feeding resulting in higher Proteobacteria and lower Firmicutes^44^,^45^,^46^. However, likely due to the fluctuations that occur in the gut microbiota in early life, other studies examining the effect of breast milk vs. formula feeding on the infant microbiome have reported anywhere from no major differences^47^ to the reverse of those found in our studies at the phylum level^1^,^48^.

Upon assessing the Shannon diversity of the groups, we found that MM fed pups displayed increased microbial species richness and evenness when compared to CTL formula. While formula feeding has previously been reported to increase alpha-diversity more than breastfeeding in the first months of life^44^,^45^,^47^,^48^,^49^, this result may reflect that within our model, formula fed pups are separated from their mother at pn day 5 and no longer exposed to maternal microbes. Of note, however, is that the MFGM formula fed pups displayed similar species richness and evenness to their MM littermates. As oligosaccharides within breast milk have been implicated in driving the diversity of the gut microbiome, components within the mammary epithelial membrane that form the outer membrane of the MFGM may also be involved. Assessment of lower taxonomic levels revealed differences in diversity between the MM, MFGM and CTL formula groups. It has previously been shown that formula feeding results in increased numbers of Clostridiales, Streptococcus, Enterococcus and Enterobacteriaceae^35^,^50^, which was also observed in this study, as these groups were higher in MFGM and CTL formula pups. Breast-feeding has been associated with increased abundance of Lactobacilli, which was highest in the MM group. Notably, Lactobacilli were also detected in the MFGM group, although at lower levels, and were absent in the CTL group.

Interestingly, MM pups had significantly higher levels of bacterial groups commonly associated with the healthy adult gut microbiota, including Lachnospiraceae, Ruminococcaceae, Blautia and Parabacteroides in addition to harboring 10-fold more intestinal microbes than formula fed littermates. This likely reflects their housing (and close contact) with their mothers, siblings, and their fecal matter. Future studies will be necessary to define the significance of MFGM mediated alterations in the gut microbiome on intestinal development and health in the long term. As breast milk has been associated with not only early protection from infection and diarrhea, but also with long lasting protection against allergies, asthma and other immune mediated diseases^10^,^12^,^13^,^14^, it is of significant interest to clarify whether MFGM supplemented formula will also have the ability to confer these long lasting effects.

To examine the role of intestinal microbes in intestinal development, an antibiotic cocktail was used to deplete gut bacteria. Surprisingly, antibiotic exposure in MFGM formula fed pups only depleted 45% of microbes, potentially due to the effects of formula delivery via gastric cannula on gut motility, or perhaps because the formula fed pups carried a smaller number of starting microbes. Due to this limited depletion in the artificially reared pups, we decided to focus our antibiotic studies on MM fed pups where antibiotic exposure led to a 10-fold depletion of intestinal microbes. Our analysis confirmed previous studies in germ free mice, that many aspects of intestinal development are partially dependent on the presence of gut microbes^51^,^52^. Upon depletion of the gut microbes within MM fed pups, significant decreases in the previously mentioned histological parameters were observed, similar to those seen in untreated CTL formula fed littermates. Interestingly, although antibiotic treatment of MM pups also resulted in similar numbers of gut microbes to that carried by untreated MFGM formula fed pups, villus lengths and crypt depths were significantly greater in the MFGM fed pups. This suggests that in addition to exerting some of its beneficial effects via changes to the microbiome, MFGM likely contributes to normalizing intestinal development through additional mechanisms, potentially acting directly on the intestinal epithelium itself.

As diarrhea and enteric infections present at higher rates in formula fed infants, we next tested whether MFGM supplementation promoted a protective effect against intra-rectal *C. difficile* toxin challenge. There is growing evidence that *C. difficile* is associated with diarrhea and increased lengths of hospital stays in children under 2 years of age^53^,^54^,^55^. *C. difficile* infections may be underreported in this population, as it is often not associated with the severe outcomes observed in infected adults such as colon resection and mortality^53^. In our neonate model, *C. difficile* toxins induced a rapid colitis, albeit to a lesser degree than previously described in adult mice. Using this model we showed that the accelerated intestinal development in MFGM formula supplemented pups was protective, leading to significantly reduced colitis as compared to the CTL formula fed group. As Engevik *et al*., have recently reported that patients with *C. difficile* associated intestinal disease exhibit decreased Muc2 and an impaired mucus barrier^56^, the ability of MFGM supplementation to increase Muc2 levels in the colon may be one mechanism by which MFGM conferred protection in this model. Furthermore, intracellular and membrane proteins within MFGM comprise 2–4% of the proteins found within breast milk, with many of them having antibacterial or immune-modulatory functions^57^. Therefore, the proteins found within MFGM may also be contributing to the protection conferred in this model.

Taken together, this study has shown that ingestion of MFGM supplemented formula by rat pups normalizes many aspects of their intestinal development to levels similar to those seen in MM pups. We have also shown, for the first time, that addition of MFGM in formula promotes intestinal epithelial cell proliferation, TJ protein patterns, and development of IEC subsets (PCs, GCs and enterocytes) to levels above that seen with CTL formula, and similar to those in MM fed pups. Interestingly, MFGM formula also impacted the intestinal microbiota of formula fed pups, leading to a microbiome more similar to that of MM pups, which likely has important implications for long-term health. Moreover, the addition of MFGM to formula protected pups against intestinal challenge with *C. difficile* toxins, as compared to the colitis suffered by CTL formula fed pups. From these results we suggest that MFGM supplementation in formula has many beneficial effects on intestinal development and promotes protection against noxious intestinal stimuli. Therefore its supplementation in formula may prove beneficial in human populations that have limited access to breast milk.

## Methods

### Animals and provision of formula

Pregnant female Sprague Dawley rats at 12 days of gestation were ordered from Charles River Laboratories (Wilmington, MA, USA). Pregnant females were housed individually in sterilized, filter-topped cages and fed autoclaved food and water under specific-pathogen-free conditions at the BC Children’s Hospital Research Institute (BCHRI). At pn day 5, rat pups from each litter were randomly assigned to three different formula supplementation groups (control formula, or formula containing either 1.2 or 6 g/L bovine MFGM), with age-matched mother milk (MM) reared littermates used as reference controls for intestinal development. In brief, gastrostomy feeding of the formula groups commenced on pn day 5 via PE 10 polyethylene tubing cannulas inserted into the stomach, as previously described in detail^42^. Pups were anaesthetized using halothane for the duration of the cannulation procedure. Gastric cannulas were connected to PE 50 tubing attached to Ismatec peristaltic pumps, which delivered volume controlled amounts of formula over 24 hours (2 cycles/hour, 10 minutes on, 20 minutes pause) to pups housed in an incubated water bath to maintain body temperature. The protocols employed were approved by the University of British Columbia’s Animal Care Committee and were in direct accordance with guidelines provided by the Canadian Council on the Use of Laboratory Animals.

### Milk fat supplementation

Cannulated rat pups were provided either a control (CTL) formula rat’s milk substitute (details of formula provided in Supplementary Table 1) reflecting the vegetable derived fat source currently available in infant formulas, or alternatively, formula supplemented with bovine Milk Fat Globule Membrane (Lacprodan MFGM-10^®^, kindly provided by Arla Foods Ingredients (Aarhus, Denmark) and Mead Johnson Nutrition (Evansville, IN, USA). MFGM concentrations used for this study were calculated based on the amount of phospholipid (PL) in breast milk, as MFGM is the sole source of PL in breast milk. Human breast milk ranges from 3–4% fat, therefore to reflect this content, a concentration of 1.2 g/L of MFGM was used. As rat milk contains a higher % of fat (13–15%) a concentration of 6 g/L of MFGM was also used, to more accurately reflect this fat content. Formula was prepared and stored at −20 °C, and thawed and mixed with a polytron prior to use.

### Tissue collection

Rat pups were anesthetized with isofluorane and euthanized via cervical dislocation from (1) MM, MFGM and CTL formula groups at pn day 15 (8–10 pups/group), (2) after 7 days of antibiotic treatment at pn d15 in the MM group along with untreated MM and CTL formula pups (5 pups/group), or (3) at pn day 15 following 2 hours of *Clostridium difficile* TcdA/TcdB toxin exposure (6–8 pups/group). For histology and immunohistochemical staining, jejunal, ileal and distal colonic samples were fixed in 10% neutral buffered formalin (Fisher Scientific, Waltham, MA, USA) or Carnoy’s solution (60% ethanol, 30% chloroform, 10% glacial acetic acid). Samples were allowed to fix overnight (formalin) or for 2 hours (Carnoy’s) at 4 °C, transferred to 70% or 100% ethanol respectively, embedded in paraffin and cut into 5-μm sections. Tissue sections with >50% of cross section intact on slides were included in immunofluorescent analysis, resulting in 3–4 images per tissue section assessed for 6–10 tissues per group. Stool samples were also collected for intestinal microbe analysis.

### Crypt depth and villus height measurements

Hematoxylin and eosin stained paraffin sections from the jejunum, ileum, and colon were viewed under brightfield on a Zeiss Axio Imager microscope as outlined previously^31^. Villus heights and crypt depths were measured in images using the measurement tool on AxioVision software at 200X magnification. At least 10 height or depth measurements were made per tissue section imaged.

### FITC-dextran barrier permeability assay

Barrier permeability was assessed as previously described^34^,^58^. Briefly, rat pups were gavaged at pn day 15 (4 hours before euthanization) with 12 mg of 4 kDa FITC (fluorescein isothiocyanate)-dextran in PBS. At sacrifice, blood was collected by cardiac puncture and added to 3% acid-citrate dextrose. FITC-dextran concentration in serum was measured using a fluorometer (excitation λ 485 nm, emission λ 530 nm).

### Immunofluorescence

Paraffin embedded sections (~5-μm thick) were deparaffinized by heating to 60 °C for 15 min, cleared with xylene, followed by an ethanol gradient (75%, 95%, 100%) and water and steamed for 30 min in citrate buffer for antigen retrieval. Tissues were then treated with blocking buffer (goat or donkey serum in PBS containing 1% bovine serum albumin [BSA], 0.1% Triton X-100, 0.05% Tween 20, and 0.05% sodium azide). The primary antibodies used were anti-Ki-67 (Thermo Scientific, Waltham, MA, USA), anti-Lysozyme (Santa Cruz, Dallas, TX, USA), anti-Muc2 (Santa Cruz), anti-CA-1 (Santa Cruz), anti-5HT (Antibodies Incorporated, Davis, CA, USA), anti-Claudin-3 (Invitrogen, Carlsbad, CA, USA), anti-Claudin-4 (Invitrogen). The secondary antibodies used were Alexa Fluor 568- or 488-conjugated goat anti-rabbit IgG, and Alexa Fluor 568- or 488-conjugated donkey anti-rabbit or donkey anti-goat IgG (Life Technologies, Carlsbad, CA, USA). ProLong gold antifade reagent with 4′,6-diamidino-2-phenylindole (DAPI; Invitrogen, Carlsbad, CA, USA) to stain DNA was used to mount tissues. Tissues were viewed on a Zeiss Axio Imager microscope, and images were taken using AxioVision software and an AxioCam HRm camera.

### Fluorescence intensity measurements

To semi-quantify positive staining in tissue sections, the fluorescence intensity of immunostained samples was assessed using the integrated density measurement tool on ImageJ software. The fluorescence intensity was represented as the integrated density value of Muc2 or CA-1 (small intestinal sections only) relative to total DAPI values per tissue section or fluorescence intensity of Claudins or CA-1 (colonic sections only) in villi relative to crypts (small intestinal sections) and at top of crypts relative to the rest of crypts in colonic sections. Note that slides for all three groups from each experiment were stained at the same time and under the same conditions.

### Intestinal microbe counts

Total microbes in the colonic luminal contents were enumerated as previously described^31^,^58^. In brief, following euthanization, colonic contents were collected, weighed, and homogenized in PBS. Homogenized samples were fixed in 10% Neutral Buffered Formalin to a final concentration of 3%. Samples were further diluted 1:10 in PBS, vortexed briefly, then 5 to 10 μl of these diluted samples were diluted in PBS to a final volume of 1 mL and filtered onto Anodisc 25 filters (Whatman International Ltd, Maidstone, UK) with a pore size of 0.2 μM and 2.5 cm diameter. Filters were then mounted onto glass slides with DAPI ProLong gold antifade reagent after drying and viewed at 1000X magnification on a Zeiss Axio Imager microscope. The number of DAPI positive microbes was counted in 3 to 6 randomly chosen fields per disc, by two blinded scorers. The total number of intestinal microbes was calculated based on the mean of all counted fields per sample and the dilution factor used.

### DNA extraction and 16S rRNA gene sequencing, processing and analysis

Bacterial DNA extractions were performed as previously described in detail^59^,^60^. Briefly, colonic contents were mechanically homogenized with glass beads (MoBio, Carlsbad, CA, USA) in 200 mM NaPO_4_, pH 8 and guanidine thiocyanate-EDTA-*N*-lauroyl sarcosine. Enzymatic lysis was performed for 1 h at 37 °C with 50 μl lysozyme (100 mg/ml), 50 μl mutanolysin (10 U/μl), and 10 μl RNase A (10 mg/ml), followed by another incubation for 1 hr at 65 °C after addition of 25 μl 25% SDS, 25 μl Proteinase K (20 mg/ml,) and 75 μl 5 M NaCl. Supernatants were collected and DNA extracted with phenol-chloroform-isoamyl alcohol (25:24:1; Sigma, St. Louis, MO, USA) and purified using DNA Clean and Concentrator-25 columns (Zymo, Irvine, CA, USA) as per manufacturer’s instructions (purified DNA was stored at −20 °C).

PCR amplification of the V3 region of the 16S rRNA gene was performed as previously described^61^, with the following modifications: a 50 μl reaction containing 1.25 mM MgCl_2_, 2.5 mM of each dNTP, 100 nM of each barcoded primer, and 0.25 U Taq was divided into 3 × 20 μl reactions for amplification. PCR conditions consisted of an initial denaturation at 94 °C for 2 min, 25 cycles of 94 °C for 30 s, 50 °C for 30 s, 72 °C for 30 s, followed by a final elongation at 72 °C for 10 min. Purified PCR products were sequenced using the Illumina MiSeq platform at the McMaster Genome Facility (Hamilton, ON, Canada). 16S rRNA gene sequence processing was completed as previously described^60^,^62^,^63^. Sequences were filtered for quality with Sickle using a threshold of 30^64^ and chimera checking was performed using USEARCH^65^. OTUs were binned at 97% similarity using AbundantOTU^66^ and taxonomy was assigned using the Ribosomal Database Project (RDP) classifier^67^ against the Greengenes reference database (4 February 2011 release)^68^ using Quantitative Insights Into Microbial Ecology (QIIME)^69^. Unassigned OTUs and singletons were not included. The total number of sequence reads after filtering was 766, 232 (average 51, 082 reads/sample; range: 25, 673–71, 590 reads). Relative abundance taxonomic summaries, alpha diversity and beta diversity were performed using QIIME.

### Antibiotic treatments

Rat pups (MM reared) were treated with an antibiotic cocktail daily from pn day 8 through to 15 by oral gavage. The cocktail consisted of 1 g/L ampicillin sodium, 1 g/L neomycin sulfate, 1 g/L metronidazole and 0.5 g/L vancomycin hydrochloride administered at a volume of 10 mL/kg body weight. Crypt depths, villus heights, Paneth cell numbers, Ki-67 positive cells and Muc2 fluorescence intensity for antibiotic treated groups were expressed relative to their untreated groups, as described above.

### *Clostridium difficile* toxin induced intestinal inflammation

*C. difficile* toxins TcdA and TcdB were kindly provided by Dr. Paul Beck, University of Calgary (Calgary, AB, Canada). Intra-rectal instillation of *C. difficile* toxins was performed as previously described^37^,^38^. Briefly, a PE-50 polyethylene catheter was attached to a 19G needle on a 1 mL syringe and lubricated with a water-soluble lubricant. The catheter was inserted 2.5 cm into the colon of anesthetized pups, and 50 μg TcdA/TcdB toxin in 100 μL PBS was slowly injected over 30 seconds. Upon removal of the catheter, pressure was applied to the anal area for a further 30 seconds to prevent the diluted toxin from leaking out. Histological damage was scored by 2 blinded observers using the following scoring criteria: (a) architectural changes: 0 = normal, 1 = vacuolation/blebbing, 2 = loss of epithelium, 3 = complete loss of crypt architecture; (b) inflammatory cell infiltrate: 0 = normal, 1 = increased inflammatory cell infiltrate in lamina propria, 2 = inflammatory cell infiltrate in submucosa, 3 = dense collection of inflammatory cells, but not transmural, 4 = transmural inflammatory cell infiltrate; (c) goblet cell depletion: 0 = no change, 1 = <50% depletion, 2 = >50% depletion, 3 = >90% depletion. For each section assessed, parameters (a) and (b) were multiplied by the percentage of the colonic tissue section exhibiting changes to more accurately reflect the damage for each tissue.

### Statistical Analysis

Results presented in this study, unless otherwise noted, were expressed as the mean value ± standard error of the mean (SEM) and analyzed using GraphPad Prism 6 for Mac OS X. Shapiro-Wilk tests were performed on all groups, where possible, to determine normality prior to analysis. Differences between means were calculated using Student’s t-tests or analysis of variance (ANOVA) (one- or two- way), followed by a *post hoc* Tukey test where appropriate. If normality was not achieved, non-parametric Kruskal-Wallis or Mann-Whitney tests were performed to determine differences.

Statistical significance of phyla abundance was determined using ANOVA with Tukey’s multiple comparison test, while alpha diversity significance was determined using Kruskal-Wallis with Dunn’s multiple comparison. Statistically significant OTUs between MM, MFGM, and CTL groups were identified using LEfSe^70^. Adonis, implemented in QIIME, was used to determine statistical significance of groups using beta-diversity distances. A *p* value of <0.05 was considered statistically significant.

## Additional Information

**How to cite this article:** Bhinder, G. *et al*. Milk Fat Globule Membrane Supplementation in Formula Modulates the Neonatal Gut Microbiome and Normalizes Intestinal Development. *Sci. Rep.* **7**, 45274; doi: 10.1038/srep45274 (2017).

**Publisher's note:** Springer Nature remains neutral with regard to jurisdictional claims in published maps and institutional affiliations.

## Supplementary Material

Supplementary Figures
