# Supplementary material for: Milk Fat Globule Membrane Supplementation in Formula Modulates the Neonatal Gut Microbiome and Normalizes Intestinal Development
Source: Sci Rep. 2017 Mar 28;7:45274. doi: 10.1038/srep45274 (PMC5368573; doi:10.1038/srep45274)
Supplement: Supplementary Figures [file srep45274-s1.pdf]

Milk Fat Globule Membrane Supplementation in Formula Modulates the Neonatal Gut Microbiome and Normalizes Intestinal Development

Ganive Bhinder[1], Joannie M. Allaire[1,] Cyrielle Garcia[2#], Jennifer T. Lau[3], Justin M. Chan[1], Natasha R. Ryz[1], Else S. Bosman[1], Franziska A. Graef[1], Shauna M. Crowley[1], Larissa S. Celiberto[1], Julia C. Berkmann[1], Roger A. Dyer[2], Kevan Jacobson[1], Michael G. Surette[4], Sheila M. Innis[2§‡]and Bruce A. Vallance[1§*]

Supplementary Materials

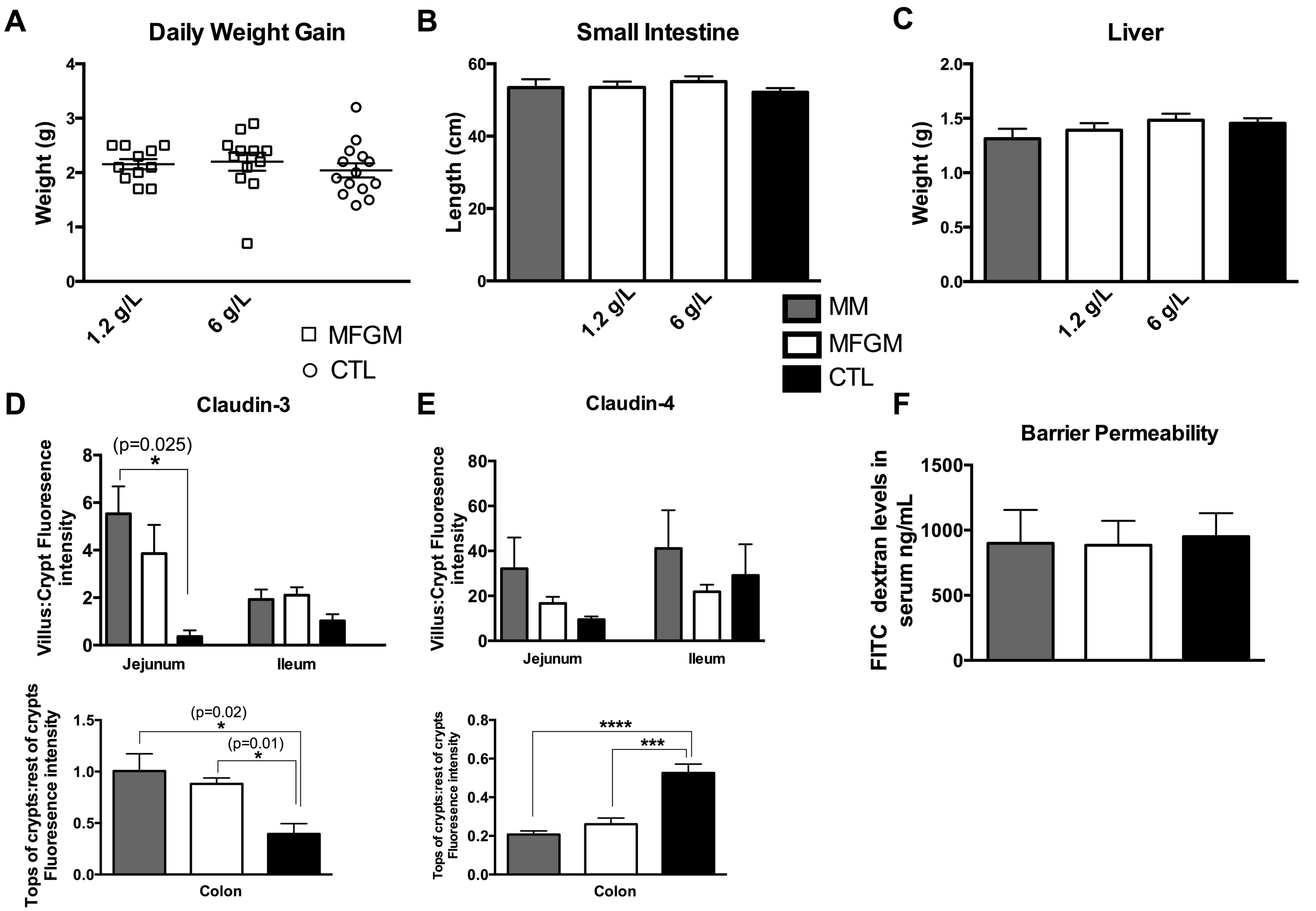

Supplementary Figure 1: Formula feeding does not alter overall growth of rat pups compared to MM fed by pn day 15. (A) Average daily weight gain from pn day 5 to 15 did not differ between pups supplemented with CTL formula, 1.2 g/L or 6 g/L MFGM. n>10. Small intestinal length (B) and liver weight (C) were similar in the four groups at pn day 15. n>10 (D) Claudin-3 and (E) Claudin-4 fluorescence intensity ratios in villi relative to crypts in small intestinal sections (top panels) and intensity at top of crypts relative to the rest of crypts (bottom panels) at pn day 15, n>6. (F) Assessment of intestinal barrier integrity using the FITC-dextran barrier permeability assay revealed similar permeability between MM, 6 g/L MFGM and CTL formula fed pups. n>4. The graphed data presented are the mean ± SEM, analyzed by One-way ANOVA followed by post hoc Tukey's test, *p<0.05, ***p<0.0005, ****p<0.0001.

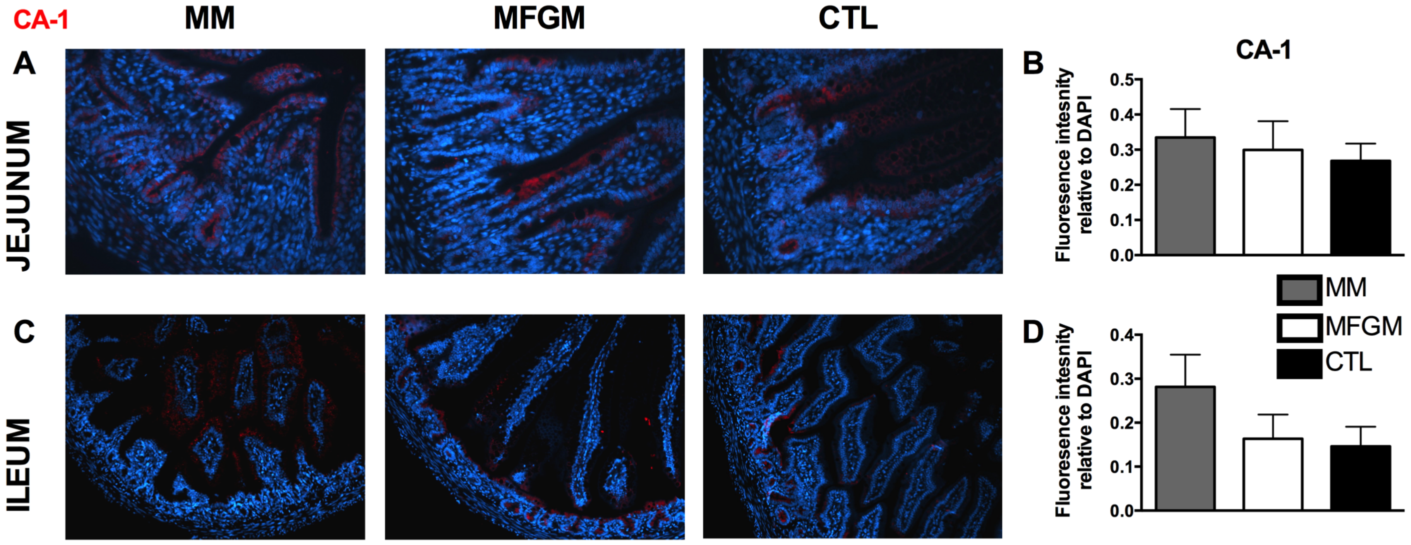

Supplementary Figure 2: MM, 6 g/L MFGM and CTL formula fed pups display similar CA-1 staining at small intestinal sites. Representative immunostaining of enterocyte marker CA-1 (red) and DNA (blue) in the jejunum (A) and ileum (C) of pups at pn day 15, semi-quantified for fluorescence intensity of CA-1 relative to DAPI in (B) and (D). n>5. The graphed data presented are the mean ± SEM. Original Magnification: 200X.

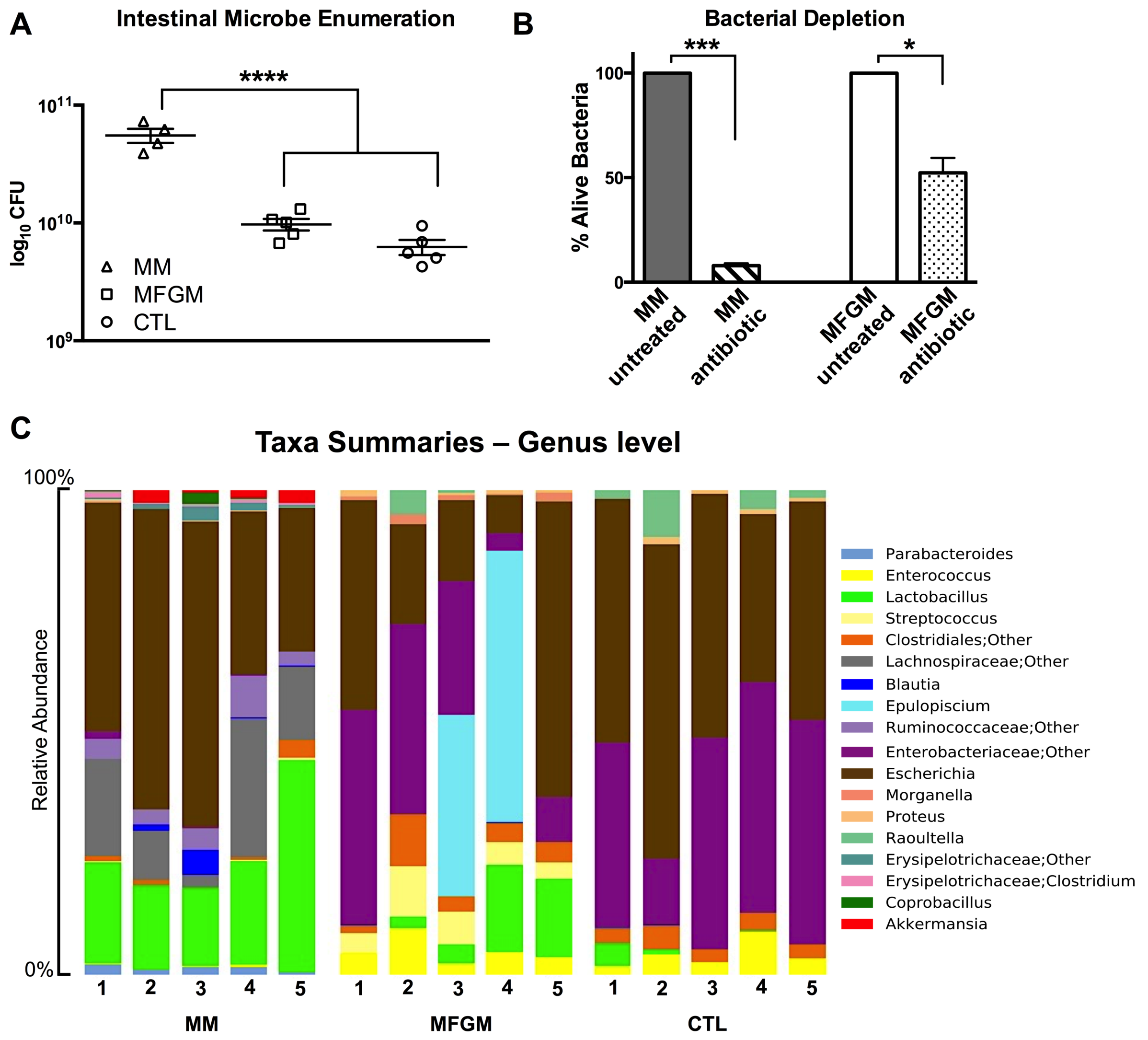

Supplementary Figure 3: MM fed pups have significantly greater intestinal microbes compared to formula fed littermates, which can be efficiently depleted with antibiotic exposure. **(A)** Intestinal microbe numbers were assessed in colonic luminal contents of MM, 6 g/L MFGM and CTL formula fed pups at pn day 15, with MM pups harbouring ten-fold greater microbes than formula fed groups. The graphed data presented are the mean ± SEM, analyzed by One-way ANOVA. n=5 **(B)** Daily oral gavage of 10 mL/kg antibiotic cocktail from pn day 8 to 15, led to a significant decrease (>90%) of intestinal microbes in MM fed pups, compared to approximately 45% depletion in 6 g/L MFGM fed pups. The graphed data presented are the mean ± SEM, analyzed by unpaired Student's t-test. n=5 **(C)** Assessment of relative abundance of microbes at the genus level in fecal pellets using 16S rRNA gene sequencing at pn day 15. *$p<0.05$, ***$p<0.0005$, ****$p<0.0001$.

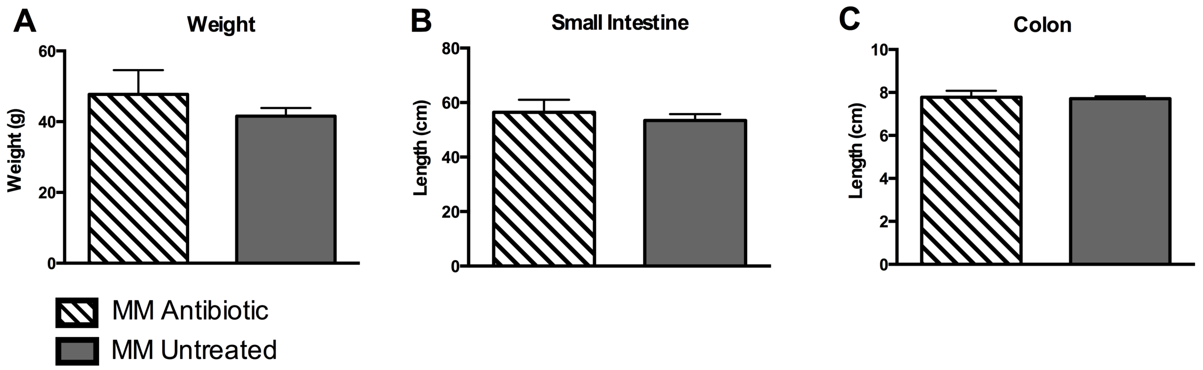

Supplementary Figure 4: Daily antibiotic exposure does not alter overall growth of MM pups compared to untreated MM littermates. Weight **(A)**, small intestinal length **(B)** and colon length **(C)** are similar between antibiotic treated (pn day 8 to 15) and untreated MM fed pups at pn day 15. The graphed data presented are the mean ± SEM, analyzed by unpaired Student's t-test. n=5

Nutrient	Amount in 1 L formula
Casein	70 g
Whey	47 g
Lactose	30 g (in 120 mL H_2O)
Basic Buffer (0.513 g NaOH, 1.5 g KH_2PO_4, 9.0 g AIN93G mineral mix (Teklad), 317 mL H_2O)	317 mL
Carnitine (0.01 g/mL)	400 µL
Creatine (0.01 g/mL)	7 mL
Ethanolamine	0.0342 g (33 µL)
Amino Acid Mix (25 g Arginine, 15 g Glycine, 7.5 g Taurine, 1.0 g Picolinic Acid)	0.97 g
Vitamin mix (Teklad 40060)	4.0 g
Supplementary Vitamin mix (0.4175 g Riboflavin, 0.65 g Niacin, 0.3475 g Pyridoxal, 23.24 g Inositol)	0.55 g
Fat Mix (20 g Coconut oil, 41 g Corn oil, 26 g Soy oil, 53 g Captex MCT)	140 g
Additional Fat Added:	
CTL formula	6.36 g/L Soy oil
MFGM formula (1.2 g/L)	5.16 g/L Soy oil, 1.2 g/L MFGM powder
MFGM formula (6 g/L)	0.36 g/L Soy oil, 6 g/L MFGM powder

Supplementary Table 1: Composition of Formulas. Amounts are listed per 1L of formula preparation.
